# Flowering process in soybean under water deficit conditions: A review on genetic aspects

**DOI:** 10.1590/1678-4685-GMB-2021-0016

**Published:** 2021-12-13

**Authors:** Mayla Daiane Correa Molinari, Renata Fuganti-Pagliarini, Daniel de Amorim Barbosa, Silvana Regina Rockenbach Marin, Daniel Rockenbach Marin, Elíbio Leopoldo Rech, Liliane Marcia Mertz-Henning, Alexandre Lima Nepomuceno

**Affiliations:** 1 Universidade Estadual de Londrina, Departamento de Biologia Geral, Londrina, PR, Brazil.; 2 Embrapa Soja, Londrina, PR, Brazil.; 3 Embrapa Recursos Genéticos e Biotecnologia, Instituto Nacional de Ciência e Tecnologia em Biologia Sintética, Brasília, DF, Brazil.

**Keywords:** Water deficit, Glycine max, climate changes, flowers, pods

## Abstract

Soybean is a key crop in many countries, being used from human food to the animal industry due to its nutritional properties. Financially, the grain chain moves large sums of money into the economy of producing countries. However, like other agricultural commodities around the world, it can have its final yield seriously compromised by abiotic environmental stressors, like drought. As flowers imply in pods and in grains inside it to minimize damages caused by water restriction, researchers have focused on understanding flowering-process related genes and their interactions. Here a review dedicated to the soybean flowering process and gene network involved in it is presented, describing gene interactions and how genes act in this complex mechanism, also ruled by environmental triggers such as day-light and circadian cycle. The objective was to gather information and insights on the soybean flowering process, aiming to provide knowledge useful to assist in the development of drought-tolerant soybean lines, minimizing losses due to delays or anticipation of flowering and, consequently, restraining financial and productivity losses.

## Introduction

Soybean is one of the most planted commodities in the world. It is a source of animal and human food due to its various nutrient and functional compounds. Also, the grain is widely used in the oil and animal feed industry, employing thousands of people, and moving millions of dollars annually. From 2015 to 2019, the USA was in the first place in the ranking of global soybean producers (USDA, 2021). In 2020, Brazil outstripped the United States, producing 124 million metric tons, production about 3% higher than the USA, taking the leadership position in the rank of producers and remaining as a main player in 2020/2021 crop season with about 137 million metrics (CONAB, 2021; USDA, 2021).

Despite high productivity, in crop seasons affected by abiotic conditions such as drought, yield can be seriously impaired, leading to significant economic losses. If the lack of water occurs in sensitive developmental periods such as in flowering and pod filling, the impact can be huge, downsizing productivity between 78 to 97% when compared to good-water crop seasons (Ferreira, 2016). Data collected from 1976/77 to 2013/14 crop seasons in Brazil indicated up to US$79,62 billion in financial losses due to water deficit (Ferreira, 2016). 

Thus, drought can jeopardize the main hope of any soybean producer, which is to have his crop filling as many pods as possible. However, it begins with the number of nodes followed by the number of flowers set. The greater the number of nodes and branches, the greater the flower-bearing potential. In other words, the more physically spread-out flowers are on a plant, the greater the final production of pods. However, under drought conditions, flowers and pods abortion can occur, dropping final yields numbers. In pods, the period of latter pod formation is particularly critical, as flowering has ceased, there is no further compensation for lost pods. Usually, vulnerability to abortion under water deficit is higher in younger pods when compared to older pods/seeds. Depending on soybean genetics, seed size can still be compensated, if rain occurs after R5 (pod fill), which can reduce yield losses. Furthermore, seed number per pod and seed size can also be impaired but to a minor magnitude than pod numbers (Desclaux et al., 2000). Water deficit-stressed plants frequently mature earlier, shortening the grain filling period, and consequently reducing seed weight and final yield (Licht et al., 2013). 

Therefore, considering the current climate scenario and future projections pointing out that the type, frequency, and intensity of extreme events should increase as Earth’s climate changes (IPCC, 2021), the comprehension of the flowering mechanisms and the genes involved in this pathway, could give necessary information and be an alternative tool to minimize losses, in the next decades, since the switching from the vegetative to the reproductive phase relates closely to the success in crop productivity (Cai et al., 2020). Moreover, with the currently available editing tools, these genes can be candidates in the development of new soybean cultivars aiming to eliminate or mitigate drought- stress, ensuring that a great number of flowers/pods would survive to reduce yield losses. 

## Gene network involved in flowering in soybean - studies involving mutation and/or overexpression

Soybean is a short-day dicot plant (i.e., long nights or dark periods for flowering induction), meaning that the cue for floral induction depends on soybean leaves’ capacity to assess the night length (from dusk to dawn). Therefore, the flowering process starts after a plant is exposed to a few successive nights longer than the critical day length. At this point, unifoliolate leaflets appear at stem node 1 (vegetative developmental phase V1 (leaves fully developed unifoliolate) and a young trifoliolate leaf appears at the second node. The induction continues after that in every consecutive leaf (Fehr and Caviness, 1977; Wilkerson et al. 1989). 

Due to this short-day plant characteristic, the soybean genetic improvement process carried out over the years, especially in the ‘70s, through the manipulation of genes involved in flowering, using classical breeding methodologies, allowed its growth and development in several areas of the world and in different regions of producer’s countries (Neumaier et al., 2000; Cai et al., 2020), either by delaying or advancing flowering, according to the geographical conditions (Cao et al., 2017; Cai *et al*., 2020). To be cultivated at higher latitudes, soybean breeders reduced the soybean sensitivity to the photoperiod, generating cultivars with a longer juvenile period (Farias et al., 2007; Lu et al., 2017). 

Photoperiod sensitivity is genotype-dependent, and the response degree to the photoperiodic stimulus is a critical determinant of the adaptation region of a given crop. In sensitive soybean cultivars, the response to photoperiod is quantitative, which means that flowering will happen eventually, regardless of stimuli. Nevertheless, the period required for this will depend on the day length; on short-days, the induction is quicker than on long-days (Rodrigues et al., 2001). It is noteworthy that the too early flowering and early ripening of the soybean crop generally results in extremely low grain yields (Lu et al., 2017). 

The inclusion of the long juvenile trait in soybean cultivars prolonged the vegetative phase and increased yield when grown under short-day conditions, which allowed the cultivation of the grain into the tropical regions and sowing seasons (Lu et al., 2017). Additionally, besides flowering time, the final yield in soybean is also related to plant architecture, including leaves, stem, branches, inflorescences, and pods in each node. So, to produce high-yield soybean varieties, coordination between the vertical growth, and branching is necessary (Pedersen and Lauer, 2004). For this reason, the manipulation of genes involved directly and indirectly in the flowering pathways has been used to increase the productivity and adaptability of the crop.

Despite all these advances, knowledge about the molecular bases of flowering in soybean is limited, as well as its relationship with genes involved in other mechanisms activated during this stage of development. Flowering triggers and pathways are dependent on the maturity genes in the plants and controlled by the plant hormones, developmental stage, temperature, and water availability, among other factors (Fornara et al., 2010; Wu and Hanzawa, 2014; Lyu et al., 2020; Ye et al., 2020). Physiologically, two main cyclic processes play an important role in the flowering mechanisms: 1) photoperiod (day length), that is the solar 24-hour cycle of day and night and, 2) a within-plant circadian rhythm.

Currently, available information indicates that flowering-related genes in soybean can act as photoreceptors in response to blue (*CRY1 -* Cryptochrome Circadian Regulator 1*, FKF1/2 -* Flavin-binding, Kelch repeat, F-box 1 and 2, and *ZTL3 -* Zeitlupe 3) and red (*PHYB1* - Phytochrome B1 and *PHYA1/2/3 -* Phytochrome A1, A2, and A3) (Zhang et al., 2008; Wu et al., 2011; Xia et al., 2012; Xue et al., 2012) lights, acting as transcription factors (*FT2a -* Flowering Locus T 2a*, FT5a -* Flowering Locus T 5a*, FDL19 -* Flowering Locus D9*, LFY2 -* Leafy 2*, SOC1 -* Suppressor of overexpression of CO1*, AP1a -* Apetala 1*, SVP1 -* Short vegetative phase 1*, FLC -* Flowering Locus C*,* and *E1lb -* E1 like-b protein), regulating the expression of downstream genes by binding to conserved cis-elements (Chi et al., 2011; Na et al., 2013; Su *et al*., 2013; Nan et al., 2014; Zhang *et al.*, 2016; Zhu et al., 2018; Cai et al., 2020; Molinari et al., 2021); acting directly in the flowering process, such as *VRN1* - Vernalization 1, *ELF4* - Early Flowering 4, *COL1a/b* - Constans like 1a and 1b, and *FT4 -* Flowering Locus T 4 genes (Watanabe et al., 2011; Zhai et al., 2014; Cao et al., 2015; Suo et al., 2016; Marcolino-Gomes et al., 2017) and yet in epigenetic regulations (*FLD* - Flowering Locus D) (Hu et al., 2014). 

To identify correlations between *Arabidopsis thaliana* and soybean, Jung et al. (2012) performed an analysis of gene orthology between species. After analyzing 183 genes of *Arabidopsis thaliana* with well-established roles in the flowering process, these authors identified 491 orthologs genes in soybean. This is explained by the two duplication events that the entire soybean genome underwent during its evolution, resulting in several copies of homologous genes, which were genes with only one copy in *Arabidopsis* (Schmutz et al., 2010). 

Besides, another study crossed data from gene orthology with transcriptional analyzes in various soy tissues, including flowers and vegetables, under normal growing conditions. Results provided a set of genes involved in the flowering process that would possibly have similar responses in soybean (Libault et al., 2010; Severin et al., 2010).

**Figure 1 -:**
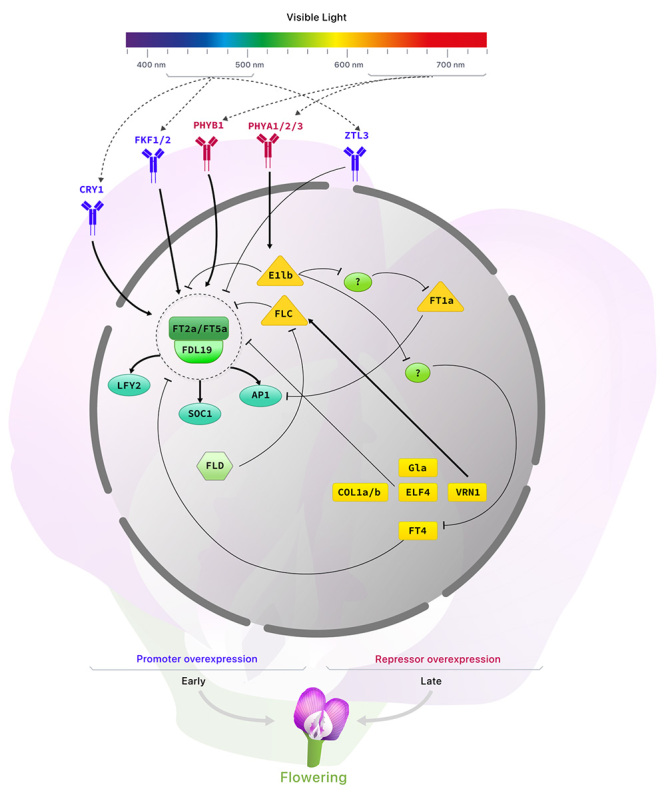
Flowering controlling pathways in soybean. Straight end lines represent repression and arrows represent activation. Red: red-light photoreceptors. Blue: blue-light photoreceptors. Shades of green: flowering promoters. Shades of yellow: flowering repressors. Gray spaced circle represents the nucleus. Dotted small circle represents the central flowering complex. Dotted arrows represent the light spectrum absorbed by the photoreceptors.

Other studies had focused on the overexpression and/or loss of gene function to access the function of these genes involved in the soybean flowering process (Table 1). Some authors inserted soybean genes in species such as Arabidopsis and tobacco, while others worked with soybean endogenous genes (cisgenesis). Table 1 summarizes the characteristics of these genes, such as phenotype related to flowering, their gene class, how their role was validated, and references. It is important to highlight that a common phenotype observed in the overexpression of a repressor gene is late flowering, while the overexpression of other genes (not repressors) resulted in early flowering. All these genes act together to regulate flowering, and some of these interactions can be seen in Figure 1.

**Table 1 -:** Flowering genes in soybean.

GENE ID	Genome Wm82.a1.v1	Genome Wm82.a2.v1	Annotation	Validation	Phenotype	Relation with	Class	Action	Citation
FDL19	Glyma19g30230	Glyma19G122800	bZIP Transcription Factor FDL19	*Gm* Overexpression in Soybean	Early flowering	Photoperiod	Transcription factor	PROMOTER	Nan et al. (2014)
FT2a	Glyma16g26660	Glyma.16G150700	Protein flowering locus T 2a	*Gm* mutant in Soybean	Late flowering	Photoperiod	Transcription factor	PROMOTER	Cai et a*l*. (2020)
FT5a	Glyma16g04830	Glyma.16G044100	Protein flowering locus T 5a	*Gm* mutant in Soybean	Late flowering	Photoperiod	Transcription factor	PROMOTER	Cai et al. (2020)
AP1a	Glyma16g13070	Glyma.16G091300	Apetala-like 1	*Gm* Overexpression in Tabaco	Early flowering	Photoperiod	Transcription factor	PROMOTER	Chi et al. (2011)
SOC1	Glyma18g45780	Glyma.18G224500	Suppressor of overexpression of CO 1	*Gm* Overexpression in Soybean	Early flowering	Circadian clock /Photoperiod	Transcription factor	PROMOTER	Na et al., (2013)
SOC1-like	Glyma09g40230	Glyma.09G266200	Suppressor of overexpression of CO 1	*Gm* Overexpression in Soybean	Early flowering	Circadian clock /Photoperiod	Transcription factor	PROMOTER	Na et al., (2013)
LFY2	Glyma06g17170	Glyma.06G163600	Protein leafy	Up-regulation in GM soybean Overexpressing *GmFDL19*	Early flowering	Photoperiod	Transcription factor	PROMOTER	Nan et al. (2014)
FT1a	Glyma18g53680	Glyma.18G298900	Protein flowering locus T 1a	*Gm* Overexpression in Soybean	Late flowering	Photoperiod	Transcription factor	REPRESSOR	Liu et a*l*. (2018)
E1b-like	Glyma18g22670	Glyma.04G143300	at1g16640 b3 domain	Soybean mutant	Early flowering	Photoperiod	Transcription factor	REPRESSOR	Zhu et al. (2018)
FLC	Glyma05g28130	Glyma.05G148700	Flowering locus C	Down-regulation in *Arabidopsis* GM Overexpressing *GmFLD*	Early flowering	Autonomous Temperature	Transcription factor	REPRESSOR	Hu et al. (2014)
SVP1	Glyma01g02880	Glyma.01G023500	Short Vegetative Phase 1	*Gm* Overexpression in Tabaco	Early flowering	Photoperiod	Transcription factor	REGULATOR	Zhang et al. (2016)
CRY1	Glyma04g11010	Glyma.04G101500	Cryptochrome 1	*Gm* Overexpression in *Arabidopsis*	Early flowering	Circadian clock /Photoperiod	Blue-light photoreceptor	PROMOTER	Zhang et al. (2008)
FKF1	Glyma05g34530	Glyma.05G239400	Flavin-binding kelch repeat f-box protein 1	*Gm* Overexpression in *Arabidopsis*	Increases flowering	Circadian clock /Photoperiod	Blue-light photoreceptor	PROMOTER	Li et al. (2013)
FKF2	Glyma08g05130	Glyma.08G046500	Flavin-binding kelch repeat f-box protein 1	*Gm* Overexpression in *Arabidopsis*	Increases flowering	Circadian clock /Photoperiod	Blue-light photoreceptor	PROMOTER	Li et al. (2013)
ZTL3	Glyma15g17480	Glyma.15G162300	Zeitelupe 3	*Gm* Overexpression in *Arabidopsis*	Late flowering	Photoperiod/Age	Blue-light photoreceptor	REPRESSOR	Xue et al. (2012)
PHYB1	Glyma09g03990	Glyma.09G035500	phytochrome b (phyb)	*Gm* Overexpression in *Arabidopsis*	Early flowering	Photoperiod	Red-light photoreceptor	PROMOTER	Wu et al. (2011)
PHYB1	Glyma15g14980	Glyma.15G140000	phytochrome b (phyb)	*Gm* Overexpression in *Arabidopsis*	Early flowering	Photoperiod	Red-light photoreceptor	PROMOTER	Wu et al. (2011)
PHY3A3	Glyma19g41210	Glyma.19G224200	phytochrome a (phya)	Soybean mutant	Early flowering	Photoperiod	Red-light photoreceptor	REPRESSOR	Xia et al. (2012)
PHYA1	Glyma10g28170	Glyma.10G141400	phytochrome a (phya)	Soybean mutant	Early flowering	Photoperiod	Red-light photoreceptor	REPRESSOR	Xia et al. (2012)
PHYA2	Glyma20g22160	Glyma.20G090000	phytochrome a (phya)	Soybean mutant	Early flowering	Photoperiod	Red-light photoreceptor	REPRESSOR	Xia et al. (2012)
ELF4	Glyma18g03130	Glyma.18G027500	Early flowering 4	*Gm* Overexpression in *Arabidopsis*	Late flowering	Circadian clock /Photoperiod	Gene effector	REPRESSOR	Marcolino-Gomes et al. (2017)
CO1a/b	Glyma08g28370	Glyma.08G255200	Constans-like 1	*Gm* Overexpression in Soybean	Late flowering	Circadian clock /Photoperiod	Gene effector	REPRESSOR	Cao et al. (2015)
GIa	Glyma10g36600	Glyma.10G221500	Gigantea Ia	Soybean mutant	Early flowering	Circadian clock /Photoperiod	Gene effector	REPRESSOR	Watanabe et al. (2011)
VRN1	Glyma11g13220	Glyma.11G124200	Vernalization 1	Up-regulation in GM soybean Overexpressing *AtDREB1A*	Late flowering	Photoperiod/ Temperature	Gene effector	REPRESSOR	Suo et al. (2016)
FT4	Glyma08g47810	Glyma.08G363100	Flowering locus T 4	*Gm* overexpression in *Arabidopsis*	Late flowering	Photoperiod	Gene effector	REPRESSOR	Zhai et al. (2014)
FLD	Glyma02g18610	Glyma.02G159100	Flowering locus D	*Gm* Overexpression in *Arabidopsis*	Early flowering	Autonomous	histone demethylase	PROMOTER	Hu et al. (2014)
FLD	Glyma07g09990	Glyma.07G090100	Flowering locus D	*Gm* Overexpression in *Arabidopsis*	Early flowering	Autonomous	histone demethylase	PROMOTER	Hu et al. (2014)
FLD	Glyma09g31770	Glyma.09G185800	Flowering locus D	*Gm* Overexpression in *Arabidopsis*	Early flowering	Autonomous	histone demethylase	PROMOTER	Hu et al. (2014)
FLD	Glyma06g38600	Glyma.06G250100	Flowering locus D	*Gm* Overexpression in *Arabidopsis*	Early flowering	Autonomous	histone demethylase	PROMOTER	Hu et al. (2014)
PPR37	Glyma12g07861	Glyma.12G073900	Pseudo-response regulator	Edited Soybean Overexpression	Late flowering	Circadian clock /Photoperiod	pseudo-response regulator protein	REPRESSOR	Wang et al. (2020)

Uppercase GM stands for genetically modified. Lowercase italic *Gm* stands for *Glycine max.* FLD stands for Flowering Locus D. bZip stands for Basic Leucine*Zipper*Domain. FDL 19 stands for FD-like 19. DREB1A stands for Dehydration responsive element-binding protein 1A.

In general, plants start flowering after the vegetative developmental phase. During this process, called floral induction, the apical meristem of the shoot begins to produce flowers, not leaves. The metabolic trigger for flowering is controlled by a complex regulatory network that monitors the environmental changes, ensuring that floral induction happens under appropriate conditions, maximizing reproductive success through seed production (Fornara et al., 2010). Light is one of the principal environmental clues to flowering, but other climate factors such as drought can modify both floral anatomy and flowering time (Liscum et al., 2003; Fang et al., 2019; Luccioni et al., 2019).

Molecular studies in soybean have shown that the central regulating genes for flowering, *GmFT2a* and *GmFT5a* genes, are both flowering activators (Kong et al., 2010; Sun et al., 2011; Nan et al., 2014; Cai et al., 2020) and *GmFT1a* (Flowering Locus T 1a) and *GmFT4* genes, designated as flowering repressors (Zhai et al., 2014; Liu et al., 2018). These genes, regulated by the *GmE1lb* transcription factor (Zhai *et al*., 2014), are responsive to the photoperiod and the circadian cycle. Also, according to phylogenetic analyzes of its protein sequence, transcription factor *GmE1lb* is specific to legumes (Xia et al., 2012) and its overexpression in soybean increased the expression levels of *GmFT4* and *GmFT1a* genes by suppressing unknown repressors of these genes (as shown in Figure 1). On the other hand, *GmE1lb*-overexpression repressed *GmFT2a* and *GmFT5a* expression (Zhai *et al*., 2014; Nan *et al*., 2014; Liu *et al*., 2018). 

Flowering is a critical element of regional adaptability and geographic distribution of soybean, and it is strongly controlled by temperature and photoperiod. An analysis of quantitative trait locus (QTL) mapping indicated that the *GmPRR37-* Pseudo-response regulator 37 gene, which encodes a pseudo-response regulator protein, is accountable for the main QTL qFT12-2, identified in a population of 308 RILs (Recombinant Inbred Lines) (Wang et al., 2020). These lines resulting from a cross between an early-flowering cultivar, named Heihe27 (HH27) and the late-flowering soybean cultivar Zigongdongdou (ZGDD), assayed in many environments. Sequencing comparative analysis results confirmed that cultivar HH27 presented a non-sense mutation that occasioned the loss of the CCT domain in the *Gm*PRR37 protein (Wang *et al.,* 2020). Soybean *Gmprr37*- ZGDD mutants CRISPR/Cas9-induced, growing under long-day (LD) conditions, showed early flowering (Wang *et al.,* 2020). The overexpression of the *GmPRR37* gene considerably delayed the flowering of GM soybean plants compared with WT, under conditions of long photoperiod. Furthermore, both the overexpression and the knockout of the *GmPRR37* gene in soybean revealed no important phenotypic modifications in flowering time, under short- day (SD) conditions (Wang *et al.,* 2020). Furthermore, under long-day (LD) situations, the expression of flowering-promoting FT homologs such as *GmFT2a* and *GmFT5a* was down-regulated by *GmPRR37* gene, while up-regulated the expression of flowering-inhibiting FT homolog *GmFT1a*. Haplotype’s analysis of the *GmPRR37* gene in 180 cultivars harvested across China identified natural *Gmprr37* mutants presenting earlier flowering, which allowed the cultivation of soybean at higher latitudes. This work revealed that the *GmPRR37* gene plays a key role in photoperiodic flowering and opens strategies to breed soybean cultivars adapted to specific farming systems and geographic regions (Wang *et al.,* 2020).

Furthermore, studies have pointed out that the flowering repressing genes *GmE1lb* and *GmFT4* showed suppression on short-days and, on the contrary, the stronger expression on long- days. Considering that the *GmE1lb* gene is soybean exclusive, the suggestion is that this crop developed a specific strategy to control the flowering time differing from the one observed in *Arabidopsis thaliana*. In this model plant, the overexpression of the *GmFT4* gene delays flowering by 9 days (Zhai et al., 2014). In soybean, the *GmFT4* gene may not be a direct target of *GmE1lb*, as it acts as a transcription repressor. The *GmFT4* gene is preferably induced over long-days, while the *GmFT2a/5a* gene is preferably induced over short-days. *GmFT4* and *GmFT2a/5a* genes oscillation throughout the day show an increase in expression at the beginning of dawn, a peak after 4h, a decrease at dusk, and then, a further increase, which suggests regulation by the circadian cycle (Zhai *et al*., 2014).

Besides genes already described, the *GmFT1a* acts as a floral repressor contributing to delay flowering time on soybean varieties, being a good candidate for genetic improvement to guarantee the successful implantation of high-yield germplasm in tropical environments. When analyzing the transcriptome of genetically modified soybean plants overexpressing the *GmFT1a* gene, was observed that higher expression levels of *GmFT1a* repress genes that specifying the identity of floral organs such as *GmAP1* (Liu et al., 2018). This gene in soybean can act downstream of *GmFT1a* and contribute to the flowering transition (Nan et al., 2014). Interestingly, results obtained by Liu *et al*. (2018) with the overexpression of *GmFT1a* were opposed to the regulatory pattern identified in a study performed by Nan *et al*. (2014). These authors observed that plants overexpressing flowering promoter genes such as *GmFT2a* and *GmFT5a* increased the levels of the *GmAP1* gene for specifying floral organ identity. These studies indicated that the flowering promoter gene *GmFT2a*/*5a* and flowering inhibitor *GmFT1a* gene can regulate the same set of genes in a competitive/antagonistic way that is still unclear (Liu *et al*., 2018).

Like *GmE1lb* and *GmFT4* genes, the *GmFT1a* gene is induced in long-day conditions, inhibited in short-days, and when expressed, it acts delaying soybean flowering and maturation. *GmFT1a* seems to be positively regulated by *GmE1lb*. Although *GmFT1a* acts as a transcription repressor, it may not be acting directly on *GmE1lb,* as observed in *GmFT4* (Xia et al., 2012; Zhai et al., 2014; Liu et al., 2018). Liu *et al*. (2018) proposed that *GmE1lb* acts as a “switch”, photoperiod-dependent, that positively regulates the expression of *GmFT1a* flowering inhibitory genes, but negatively regulates the *GmFT2a* and *GmFT5a* flowering genes. 

Nan et al. (2014) observed that the regulation of other flowering-related genes by floral activators, *GmFT2a,* and *GmFT5a*, occurs through their binding to the *GmFDL19* gene. The formation of this complex (*GmFT2a-GmFT5a-GmFDL19*) will act on the positive regulation of genes such as *GmAP1*, *GmSOC1*, and *GmLFY*. Thus, the suggestion is that the overexpression of this gene cascade promotes early flowering in soybean. Validation studies in tobacco have shown that *GmAP1* gene, when overexpressed, causes early flowering (Chi et al., 2011), as well as the overexpression of *GmSOC1* gene in soybean leads to early flowering as well (Na *et al*., 2013). It is noteworthy that both genes are considered activators of flowering.

Aiming to improve soybean yield, Cai et al. (2020) applied the CRISPR/Cas9 toolbox, to knock out the *GmFT2a* and the *GmFT5a* flowering genes and obtain a double *ft2aft5a* mutant. Under short-day conditions, the double *ft2aft5a* mutant bloomed about 31 days after the wild-type plants and preserved their vertical growth habit. During vegetative growth, these mutants produced significantly more nodes, leaf axis formed more branches, with an increase in the number of pods and seeds per plant, suggesting that the double *ft2aft5a* mutant has great potential to be introduced into the tropics.

Another known way of controlling the flowering induction in soybean occurs through the histone demethylase encoded by the *GmFLD* gene in the autonomous pathway. The *GmFLD* gene acts as a promoter of flowering by repressing the *AtFLC* gene (through demethylation) and activating the expression of *AtFT* and *AtSOC1* in genetically modified Arabidopsis. The *GmFLD* gene suppresses the transcription of *AtFLC* through epigenetic mechanisms of histone modification close to the transcription site, decreasing the levels of H3K4me3 (tri-methylation of the fourth lysine residue in histone H3) (Hu et al., 2014). In studies carried out by Hu *et al*. (2014), it was possible to observe that the overexpression of soybean gene *GmFLD19* in an Arabidopsis *fld* mutant rescued the late-flowering phenotype. 

Although the central point of flowering control involves transcription factors and flowering-related genes, as noted earlier, the onset of responses occurs through the perception of light by photoreceptors, which act upstream of the floral integrators *GmFT2a/5a* and *GmFT4/1a*. In Arabidopsis, both the overexpression of the endogenous blue-light photoreceptor (*AtZTL*) and its soybean analog (*GmZTL3*) generated delays in flowering in long-day conditions. Possibly, these genes have similar functions in soybean and Arabidopsis, as they are genes evolutionarily conserved, showing the same flowering-repression function (Xue et al., 2012). In *Arabidopsis thaliana*, the late flowering through overexpression of *AtZTL* occurs due to the strong reduction in transcriptional levels of *AtFT* (Kim et al., 2005).

While the overexpression of *GmZTL3* causes late flowering, the overexpression of other blue light photoreceptors such as *GmCRY1*, *GmFKF1,* and *GmFKF2* promotes earlier flowering in *Arabidopsis thaliana* (Zhang et al., 2008; Li et al., 2013). These same authors observed that the activity of these blue light photoreceptors (flowering activators) occurs through the up-regulation of the *AtFT* gene. Arabidopsis plants overexpressing *GmCRY1* showed an acceleration in flowering (Zhang *et al*., 2008), as well as overexpression of *GmFKF1/2* increased flowering in a short-day condition (Li *et al*., 2013).

Red light photoreceptors can also act as promoters (*PHYB1*) and repressors (*PHYBA1/2/3*) of flowering. While *GmPHYB1* acts by activating the *GmFT2a/5a* gene, the *GmPHYA* gene acts by activating *GmE1lb-like* (Wu et al., 2011; Xia et al., 2012; Zhang et al., 2016). Another soybean gene involved in the flowering process, according to Marcolino-Gomes et al. (2017), is *GmELF4*, responsive to the circadian cycle and the photoperiod. The overexpression of this gene in *Arabidopsis thaliana* delayed flowering when compared to wild-type plants. *GmELF4* gene altered the expression of the *AtFT* gene negatively, explaining the observed floral delay (Marcolino-Gomes *et al*., 2017). Like *GmELF4*, the *GmCOL1a/b*, *GmVRN1*, and *GmGIa* - Gigantea genes were identified as repressors of flowering. According to Cao et al. (2015), the *GmCOL1b* mutant soybeans bloom earlier than WT plants, when growing in long-day conditions; and the overexpression of the *GmCOL1a gene* delays flowering. These authors also observed that the overexpression of the *GmCOL1*a gene led to the downregulation of *GmFT2a* and *GmFT5a* genes, illustrating the activity of *GmCOL1*a gene as a flowering suppressor in soybean. In another study carried out by Watanabe et al. (2011), it was observed that the soybean *e2/e2* genotype (mutant for *GmGIa*) showed early flowering, inducing the expression of the *GmFT2a* gene, and showing the role of *GI* as a flowering repressor.

Still, in a study performed by Suo et al. (2016), the relationship of a gene involved in flowering (*GmVRN1-like*) with a drought-tolerance gene (*AtDREB1A -* Dehydration-responsive element binding 1A) was reported. The overexpression of DREB1A genes resulted in drought tolerance, and also caused delayed flowering (Kidokoro et al., 2015; Zhou et al., 2020). According to these authors, in genetically modified soy plants overexpressing this gene, the *GmVRN1* gene, homologous to Arabidopsis, was strongly induced. This fact was explained by the link of this transcription factor to DRE motifs (ACCGAC) in the promoter region of the *GmVRN1* gene, suggesting that the late flowering of genetically modified plants occurred due to the positive regulation of VRN1 in soybean, which is considered a flowering repressor. Previously, in another study conducted by the same authors, the overexpression of DREB genes caused the flowering delay by activating the floral repressor *AtFLC* in Arabidopsis (Seo et al., 2009 *apud* Suo *et al.,* 2016).

The SVP genes can also present different actions on the flowering time among species. According to Fornara et al. (2010), the *AtSVP* gene, a flowering repressor in Arabidopsis, can be associate with the *AtFLC* gene and inactivate the *AtFT* gene. The overexpression of the *MtSVP* gene from *Medicago*, for example, caused a delay in flowering in *Arabidopsis thaliana* (Jaudal et al., 2013), as well as the cisgenic overexpression of *AtSVP* (Hartmann et al., 2000); while the overexpression of soybean *GmSVP1* gene in tobacco accelerates flowering time (Zhang et al., 2016). These findings demonstrated the importance of studies on species-specific gene manipulation since the same gene can present different responses depending on the species in which it was inserted. Although the role of the *SVP* gene in soybean is not yet fully understood, in Arabidopsis, a study carried out by Wang et al. (2018) showed the correlation of this gene with drought. These authors showed that the overexpression of the *AtSVP* gene confers drought tolerance in *Arabidopsis thaliana* by regulating ABA catabolism.

All these reports show that the manipulation of genes to delay/accelerating the flowering process has been widely used to increase productivity, either by the overexpression of repressors or by mutation of flowering promoters. As other examples, the overexpression of the *AtFLC* gene, a flowering repressor, in tobacco, delayed flowering by 36 days and significantly increased biomass production when compared to wild-type plants (Salehi et al., 2005). Additionally, the mutation of flowering promoter genes such as *GmFT2a* and *GmFT5a,* in soybean*,* delayed grain flowering by 31 days and produced many more pods resulting in a substantial increase in the number of seeds (Cai et al., 2020).

## Flowering time and its relationship with drought- response

Flowering is not only an essential part of the plant’s reproductive process but also a critical developmental stage vulnerable to environmental stresses such as drought (Kazan and Lyons, 2016). Exposure to water deficit during this period can cause substantial yield losses in seed-producing plants. However, it is becoming increasingly evident that altering flowering time is an evolutionary strategy adopted by plants to maximize the chances of reproduction under diverse stress conditions, ranging from pathogen infection to heat, salinity, and drought. A better understanding of how complex environmental variables affect plant phenology is important for future genetic manipulation of crops aiming to increase productivity under a changing climate (Kazan and Lyons 2016; Molinari et al., 2021). The manipulation of different flowering genes triggered modifications in flowering time and drought adaptability. Publications showing this possibility were summarized in Table 2. The overexpression of flowering promoters for example triggers the development of plants more tolerant to drought and, the mutation of flowering repressors could give the plant the capacity to escape drought, by flowering earlier. Drought escape is an adaptive mechanism that enables plants to complete their life cycle before a drought event. Early flowering time and a shorter vegetative phase can be very important for production in conditions of terminal drought, since it can minimize exposure to dehydration during flowering and, consequently, losses in grain filling and final yield (Shavrukov et al., 2017; Molinari *et al*., 2021).

**Table 2 -:** Flowering genes in soybean and its relationship with drought-response.

GENE ID	Validation	Specie	Action	Flowering expected	Drought mechanism	Citation
FDL19	Overexpression	Soybean	PROMOTER	Early	Escape	Li et al., 2017
SOC1	Overexpression	*Arabidopsis thaliana*	PROMOTER	Early	Escape	Hwang et al., 2019
PHYB	Overexpression	*Arabidopsis thaliana*	PROMOTER	Early	Escape	Gonzalez et al., 2012
ELF4	Mutation	Soybean/*Arabidopsis*	REPRESSOR	Early	Escape	Jin et al., 2020
CRY1	Mutation	*Arabidopsis thaliana*	PROMOTER	Late	Tolerance	Mao et al., 2005
GIa	Overexpression	*Arabidopsis thaliana*	REPRESSOR	Late	Tolerance	Baek et al., 2020

Most flowering genes have had their functions in response to drought validated in *Arabidopsis thaliana*. Due to the similarity of the genes involved in the flowering process, it is possible to infer that the manipulation of soybean flowering genes can also result in the same response to drought. Among all the mentioned genes, only *SPV* has antagonistic functions among species. In Arabidopsis is a flowering repressor, and in soybean is a flowering promoter (Fornara et al., 2010; Zhang et al., 2016).

Amongst genes with the same function, in Arabidopsis, the flowering promoters *PHYB* and *SOC1,* when overexpressed, generated plants more tolerant to drought through earlier flowering process (González et al., 2012; Hwang et al., 2019). The overexpression of flowering promoter *PHYB* enhanced drought tolerance in adult plants of *Arabidopsis thaliana* by increasing stomatal sensitivity to ABA, when water becomes a scarce resource (González *et al.,* 2012). The mechanism underlying *PHYB*-enhanced drought tolerance could be the result of a higher capacity of wild-type plants to extract soil water, in comparison to *phyb* mutant. This osmotic adjustment is a typical response to drought, which can improve the chances of acquiring water from drying soil (González *et al.,* 2012). 

Similarly, according to Hwang et al. (2019), in *Arabidopsis thaliana soc1* mutant plants, a reduction in drought-escape response was observed. This data suggests that the overexpression of *SOC1* might contribute to adaptation by enabling plants to complete their life cycles under drought.

In soybean, the overexpression of the flowering promoter *GmFLD19* also generated plants more tolerant to water deficit, by shortening their life cycle. This gene is a transcription factor that belongs to the basic leucine zipper (*bZIP*) family. *bZIP* genes play an important role in the growth and developmental process, as well as response to various abiotic stresses, such as drought and, high salinity (Li et al., 2017). Besides that, the overexpressing of *GmFDL19* also causes early flowering in transgenic soybean plants (Hu et al., 2014; Li *et al.,* 2017). *GmFDL19* likewise enhanced tolerance to drought and, salt stress in soybean and, it is highly induced by abscisic acid (ABA), an important phytohormone, and polyethylene glycol (PEG 6000) (Li *et al*., 2017). The overexpression of *GmFDL19* in soybean enhanced drought and salt tolerances at the seedling stage. Moreover, the relative plant height and the relative shoot dry weight of transgenic plants were significantly higher than those of the WT, under drought. Furthermore, *GmFDL19* expression reduced the accumulation of Na+ ion content, up-regulated the expression of several ABA/stress-responsive genes in transgenic soybean and increased the activities of several antioxidative enzymes and chlorophyll content, but reduced malondialdehyde content (Li *et al.,* 2017). According to these authors, *GmFDL19* has potential to improve multiple stress tolerance in transgenic soybean lines.

In contrast, the mutation of flowering promoter *CRY1* confers enhanced drought tolerance in *Arabidopsis thaliana*, and the overexpression of *CRY1* resulted in greater water loss (Mao et al., 2005). This involvement of Arabidopsis *CRY1* promoters in drought response is largely dependent on their functions in inducing stomatal opening in response to blue light (Mao *et al.,* 2005). In soybean, it has been shown that cryptochromes *GmCRY1a* affect blue light inhibition of cell elongation. In soybean, *GmCRY1a* seems to be the more predominant regulator of photoperiodic flowering. The photoperiod-dependent circadian rhythmic expression of the *GmCRY1a* protein correlated with photoperiodic flowering and latitudinal distribution of different soybean accessions (Zhang et al., 2008).

Studies involving flowering repressors showed that the inhibition of *ELF4* in *Arabidopsis thaliana* and soybean, advances the flowering period. Besides that, it was observed that the inhibition of the flowering repressor *GmELF4,* increased drought tolerance in soybean. This gene could be manipulated to breed drought-tolerant varieties (Jin et al., 2020). In corroboration, when the *ELF4* gene was overexpressed the soybean plants, they became sensitive to drought (Jin *et al*., 2020).

Lastly, *GI*, a key regulator of photoperiod-dependent flowering and the circadian rhythm, is also involved in the signaling pathways for various abiotic stresses, like drought. The *Arabidopsis thaliana gi1* mutants are hypersensitive to drought due to the uncontrolled water loss triggered by the reduction of abscisic acid levels. This data suggested that the *GI* positively regulates diurnal ABA synthesis by affecting the expression of *NCED3 -* 9-cis-epoxycarotenoid dioxygenase 3 gene, contributing to drought tolerance (Baek et al., 2020; Molinari et al., 2020). For this reason, during drought stress, *GI* transcription is up-regulated (Han et al., 2013). These results give evidence that the overexpression of *GI* could potentially increase drought tolerance.

Finally, as a complement to all these studies, an improvement of knowledge about soybean flowering interaction pathways involved in the responses to drought, through the identification of genes differentially expressed in soybean flowers and pods under this stress condition would give more information and, new insights into the research focused on developing more drought-tolerant soybean lines, with fewer losses, as a result of flowers or pods abortion, and consequently productivity. 
